# To die or not to die: death signaling in nonalcoholic fatty liver disease

**DOI:** 10.1007/s00535-018-1451-5

**Published:** 2018-03-24

**Authors:** Yuko Akazawa, Kazuhiko Nakao

**Affiliations:** 1 Department of Pathology, Nagasaki University Graduate School of Biomedical Sciences, Nagasaki City, 852-8501 Nagasaki Japan; 2 Department of Gastroenterology and Hepatology, Nagasaki University Hospital, Nagasaki City, 852-8501 Nagasaki Japan

**Keywords:** Apoptosis, Non-alcoholic fatty liver disease, Endoplasmic reticulum stress, Free fatty acids, Nonalcoholic steatohepatitis

## Abstract

Non-alcoholic fatty liver disease (NAFLD) is an emerging liver disease worldwide. In subset of patients, NAFLD progresses to its advanced form, nonalcoholic steatohepatitis (NASH), which is accompanied with inflammation and fibrosis. Saturated free fatty acid-induced hepatocyte apoptosis is a feature of NASH. Death signaling in NASH does not always result in apoptosis, but can alternatively lead to the survival of cells presenting signs of pro-inflammatory and pro-fibrotic signals. With the current lack of established treatments for NASH, it is important to understand the molecular mechanisms responsible for disease development and progression. This review focuses on the latest findings in hepatocyte death signaling and discusses possible targets for intervention, including caspases, death receptor and c-Jun N-terminal kinase 1 signaling, oxidative stress, and endoplasmic reticulum stress, as well as epigenomic factors.

## Introduction

Non-alcoholic fatty liver disease (NAFLD) is the most common cause of liver disease in Western countries [1] and its incidence is increasing in Asian countries [2–4]. NAFLD comprises two forms: non-alcoholic fatty liver (NAFL) and non-alcoholic steatohepatitis (NASH) [5, 6]. NAFL is defined by the presence of hepatic steatosis without hepatocellular injury in the form of ballooning hepatocytes, whereas NASH is defined by the presence of hepatic steatosis plus hepatocyte injury and inflammation [6]. Although subset of NALFD patients develops NASH which potentially leads to fibrosis, cirrhosis, and hepatocellular carcinoma, there is no established pharmacological approach to treat NASH.

**Fig. 1:**
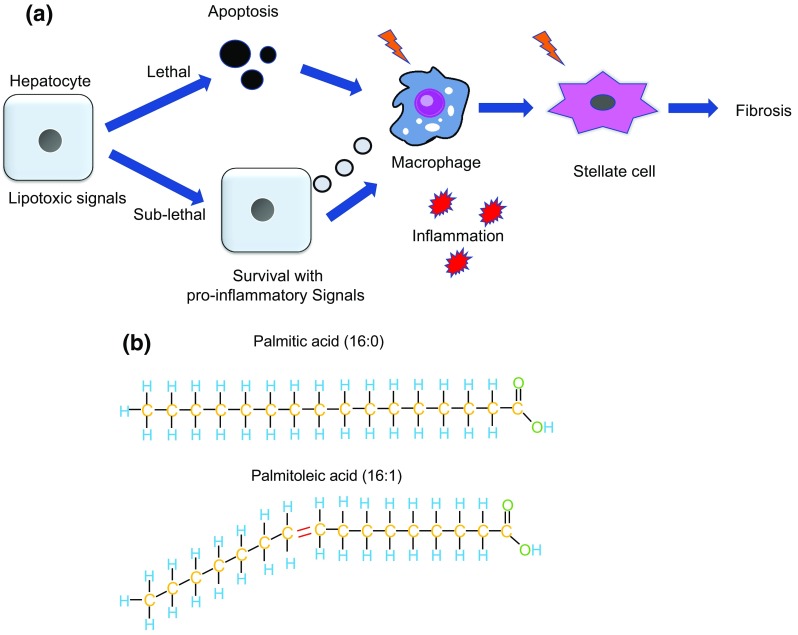
**a** Lethal and sublethal signaling during lipotoxity. Lethal lipotoxic signals induced by free fatty acids induce hepatocyte apoptosis, which are engulfed by macrophage, initiating inflammatory and fibrotic reactions. When the apoptotic signaling pathways are initiated by the apoptosis in not executed, sublethal lipotoxic signals release vesicles that are delivered to macrophages, potentially participating in progression of NASH by elevating inflammation. **b**. Simplified image of saturated free fatty acid (palmitic acid) and unsaturated free fatty acid (palmitoleic acid). Palmitoleic acid has a double bond within the carbon chain, such that when it is incorporated in the double membrane, it confers better fluidity to the membrane. Palmitic acid does not have double bonds in its carbon chain, making the membrane more rigid and less fluid

The pathogenesis of NASH is complicated and includes disruption of several sophisticated signaling networks within both hepatocytes and parenchymal cells. Emerging evidence suggests that increased hepatocyte apoptosis (termed lipoapoptosis) is a crucial mechanism that contributes to liver inflammation and fibrogenesis during NASH [7]. Consistent with this concept, apoptotic markers are increasingly recognized as indicators of NASH [8]. Dead hepatocytes are engulfed by macrophages, leading to the release of pro-inflammatory signals that activate stellate cells, ultimately inducing fibrosis (Fig. 1a).

Notably, it is becoming increasingly clear that these death signals do not always result in cell death. Rather, “sublethal” death signaling, in which the apoptotic process is initiated but not completed because only a relatively small amount of apoptotic signals is released, may lead to the activation of pathways that result in inflammation and fibrosis [9, 10] (Fig. 1a). These incomplete apoptotic signals are initiated in hepatocytes, affecting stellate cells and macrophages [11–13]. These new findings suggest potential novel targets to treat NASH. This review focuses on recent advances on lethal and sublethal hepatocyte death signals and the role they may play in the pathogenesis of NASH. In this review, lipotoxicity refers to toxicity caused by the presence of excessive free fatty acids (FFAs) and their metabolites in the cells; based on the most recent findings, it includes both sublethal and lethal effects [10, 14].

## Major toxicity-inducing agents in NASH

Long-chain fatty acids, i.e., molecules containing 12 or more linearly arranged carbon atoms, are major players in hepatocyte lipoapoptosis. In this review, FFAs generally refer to long-chain FFAs. Optimal amounts of FFAs, released mainly from subcutaneous fat by lipolysis [15], are required for membrane composition and as a source of energy. However, obesity and insulin resistance trigger adipocytes to release increased levels of circulating FFAs into the bloodstream, which then enter hepatocytes [15]. To protect themselves against lipotoxicity, hepatocytes typically induce steatosis, storing the increased amounts of FFAs as non-toxic triglycerides (TGs) [16, 17]. However, when hepatic FFAs exceed the storage limit, they activate hepatocyte death signaling [18]. Saturated FFAs are frequently found in animal fats and are toxic to hepatocytes. Saturated FFAs lack the double bond between carbon atoms and are occupied with (“saturated with”) straight hydrocarbon chains, and are solid at in vivo temperatures (Fig. 1b). Accordingly, saturated FFAs reduce membrane fluidity by making the membrane more rigid [19] and present a poor conversion into TG-enriched lipid droplets. The most common saturated FFA found in humans is palmitic acid (16:0). Unsaturated FFAs, such as oleic acid (18:1, abundant in olive oil) and palmitoleic acid (16:1, abundant in macadamia nuts) [20], are less toxic. Unsaturated FFAs possess single or multiple double bonds between their carbon atoms, giving them a “kink” in their molecular shape (Fig. 1b). Unsaturated FFAs are generally present in liquid form at biological temperatures because of their low melting temperatures and exhibit low toxicity when cultured with hepatocytes. The latter is likely due to their incorporation into TGs and the increased stability of lipid droplets containing a higher percentage of unsaturated acyl chains [16]. Omega-3 and fatty acids, such as dicosapentaenoic acid (EPA, 20:5) and docosahexaenoic acid (DHA, 22:5), as well as omega-6 fatty acids cannot be produced in humans and, thus, they are often called “essential” or “exogenous” FFAs [21]. Unsaturated FFAs counteract the toxicity of saturated FFAs, probably by increasing the fluidity of the phospholipid membrane and through incorporation of saturated FFAs into TGs [19]. In contrast, genetic or pharmacological inhibition of stearoyl-CoA desaturase-1 (SCD1), the enzyme responsible for converting saturated FFAs to mono-unsaturated FFAs, which then leads to FFA storage by TG synthesis, sensitizes cells to FFA-induced apoptosis while decreasing steatosis [22]. Furthermore, a saturated FA-rich high-fat diet (HFD) in mice leads to a more severe form of NASH than that observed in mice fed an unsaturated FA-rich HFD [23].

Although this review mainly focuses on free fatty acid-mediated lipotoxic pathways, lysophosphatidylcholine (LPC), a lipid metabolite of palmitic acid, is also cytotoxic [24]. Increased hepatic synthesis and dysregulation of cholesterol metabolism are associated with severity of NAFLD [25]. Furthermore, free cholesterol is cytotoxic, triggering hepatocyte apoptosis [26]. In addition to lipid metabolites, gut-derived bacterial endotoxins such as lipopolysaccharide (LPS) have been described as crucial cofactors in the pathogenesis of liver injury in NASH. LPS appears to induce hepatocyte apoptosis as well as inflammation, possibly by activating tumor necrosis factor alpha (TNF-α) [27]. Low doses of LPS are thought to attract neutrophil migration, further promoting hepatocyte apoptosis via the strong pro-death activity of non-parenchymal cells, especially lysosomal enzyme myeloperoxidase (MPO)-containing neutrophils [28]. Notably, LPS can also elevate tissue levels of FFA in vivo, indicating how the interaction between FFA and LPS further enhances cell death.

## Caspases: indispensable mediators of lipotoxic signaling

**Fig. 2:**
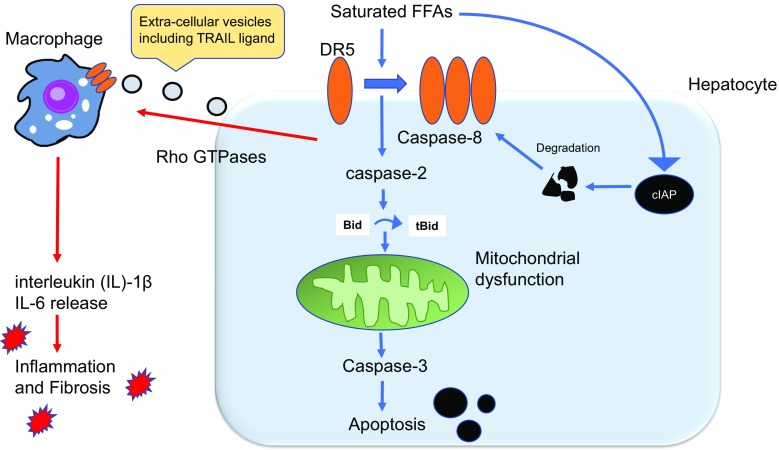
Integrated model of death receptor (DR)-mediated apoptosis and inflammation in non-alcoholic steatohepatitis (NASH). Free fatty acids (FFAs) induce aggregation of DR5 on the cell membrane and activate caspase following the formation of a complex with DR5. Caspase 8 activation results in cleavage of BH3-only protein Bid to truncated (t)-Bid, thereby contributing to mitochondrial dysfunction and cell death. Degradation of cIAP, an anti-apoptotic protein, also contributes to lipoapoptosis. Saturated FFA-induced DR5 activation also causes the release of extracellular vesicles in a Rho-GTPase-dependent manner. TNF-related apoptosis-inducing ligand (TRAIL)-containing vesicles are recognized by DR5 on macrophages, eliciting an inflammatory response and fibrosis

Caspases are a family of cysteine-proteases that execute the final phase of apoptosis. Mammalian caspases 2, 3, 7, 8, 9, and 10 are defined as apoptotic caspases, whereas caspases 1, 4, 5, 11, and 12 are associated with inflammation [29]. Caspase 3 is an indispensable caspase for chromatin condensation and DNA fragmentation, which are the final steps of apoptosis (Fig. 2) [30, 31]. As several studies have indicated caspase involvement in NASH pathogenesis, caspase inhibitors have garnered major clinical interest for the possible treatment of the disease. Several studies have suggested that pan-caspase inhibitors, including IDN-6556 (Emricasan) and VX-166, can effectively suppress apoptosis, inflammation, and fibrosis, both in vitro and in animal models [32, 33]. In addition to broad-range caspase inhibitors, it has recently been reported that specific depletion of caspase 3 protects against NASH, suggesting that targeting specific caspases is a viable approach [34]. In this particular study, caspase 3 knockout mice displayed reduced hepatocyte apoptosis and hepatic collagen deposition when fed a methionine-choline-deficient (MCD) diet.

Caspase 8 (CASP8) is an initiator caspase required for extrinsic (death receptor-mediated) apoptosis and is crucial for FFA-mediated apoptosis in hepatocytes (Fig. 2) [35, 36]. Recently, Hatting et al. employed hepatocyte-specific knockout of caspase 8 to demonstrate that a lack of this caspase decreased hepatocyte apoptosis, the expression of pro-inflammatory cytokines, and hepatic infiltration in MCD-fed mice [37]. Interestingly, although alcoholic liver diseases and NASH share similar clinical and pathological manifestations, caspase 8 inhibition does not seem to protect mice from ethanol-induced apoptosis and actually enhances caspase 9 (CASP9)-dependent intrinsic (mitochondrial) cell death by inducing release of cytochrome *c* [38]. In addition, GS-9450 is a caspase inhibitor with selective activity against caspases 1, 8, and 9, but not caspase 3. A phase 1 and 2 clinical trial of GS-9450 demonstrated an effect on chronic liver disease, including NASH [39]. In this study, significant decreases in alanine aminotransferase (ALT) and CK-18 fragments were observed in patients with NASH, suggesting that pharmacological caspase inhibitors targeting upstream death signals could also reduce hepatocyte apoptosis in NASH and might offer a valuable therapeutic strategy.

Caspase 9 is an essential initiator caspase that executes the mitochondrial pathway of apoptosis [40]. Interestingly, the ballooned hepatocyte phenotype that is a pathological feature of NASH is characterized by reduced expression of caspase 9 [12]. This is thought to be an escape mechanism from apoptosis in FA-stressed hepatocytes, as it prevents ballooned hepatocytes exposed to death signals from dying [12]. Furthermore, these cells were shown to initiate pro-fibrotic signaling through the Hedgehog signaling pathway, suggesting that ballooned hepatocytes are not solely the result of NASH but might also contribute to the development of inflammation and fibrosis [12].

Caspase 2 (CASP2) is an initiator caspase activated by various intracellular stresses and toxic agents, including saturated FFAs [29, 41, 42]. Caspase 2 was originally recognized as a mediator of mitochondrial dysfunction, promoting cytochrome *c* release from mitochondria into the cytosol (Fig. 2) [43]. Studies have reported an increased expression and FFA-induced activation of caspase 2 in patients with NASH [9]. Recent studies by Machado et al. suggested decreased apoptosis and liver injury in both caspase 2-deficient MCD diet-fed mice and a high-fat high-fructose diet-fed mouse model of NASH [9, 44]. Furthermore, caspase 2 inhibition also decreased lipotoxicity-induced Hedgehog signaling, a known mediator of fibrotic activity, as well as fibrosis [9]. Caspase 2 depletion also seems to alter the metabolic state of mice via an undefined mechanism, preventing insulin resistance and obesity [44, 45]. As caspase 2 deletion in mice caused no significant phenotype changes in the experiments of Machado et al., caspase 2 may be an attractive target for NASH treatment. However, caution is advised as a tumor-suppressive role for caspase 2 has been suggested and caspase inhibition might contribute to genomic instability and carcinogenesis in the long term [46, 47].

Taken together, both clinical and experimental data suggest that caspases are attractive candidates for the treatment of NASH. In particular, inhibition of specific caspases may enable therapeutics to focus on the disease target and reduce adverse effects. Interestingly, it has recently been shown that sublethal amounts of caspase 3, induced by FFAs, can lead to the release of pro-inflammatory vesicles from hepatocyte membranes, which can activate macrophages and may exacerbate inflammation [11, 48]. These important findings show that caspase inhibitors not only improve NASH by decreasing cell death but can also decrease inflammation when apoptosis is incomplete.

## Death receptors and ligands in NASH: an emerging role in inflammation

Hepatocyte lipoapoptosis is often triggered by death receptors (DRs) on the plasma membrane (Fig. 2) [35, 49]. In some cells, such as lymphocytes, DR activation can directly activate caspase 3. However, in hepatocytes, DR signaling requires amplification through the intrinsic mitochondrial pathway, which then leads to caspase 3 activation and cell death (Fig. 2) [35]. The major DRs include FAS, TNF receptor 1 (TNFR1), and TNF-related apoptosis-inducing ligand (TRAIL) receptors 1 and 2 (also known as DR 4 and DR5). DR5 in particular appears to play a major role in FFA-induced hepatocyte death [36]. After stimulation by FFA, DR5 undergoes self-aggregation on the plasma membrane and activates caspase 8. This cleaves the BH3-only protein BID, linking extracellular death signaling to mitochondrial dysfunction (Fig. 2) [50]. In addition, palmitic acid induces degradation of inhibitor of apoptosis protein 1 (cIAP1 or BIRC2), enhancing DR5-related signaling and lipoapoptosis (Fig. 2) [51]. RNA interference (RNAi)-based depletion of BID, a crucial player linking DR activation and mitochondrial dysfunction, attenuates NASH in a murine model [52]. Furthermore, recent studies have shown that DR5 contributes to macrophage-associated inflammation in NASH [48]. Interestingly, DR5 up-regulation by FFAs not only induces cell death, but also contributes to the release of hepatocyte-derived extracellular vesicles (EVs) responsible for intercellular communication [53]. Such vesicles are increasingly being recognized as potential factors in the pathogenesis of NASH [13, 48, 54, 55]. Consequently, TRAIL receptor inhibition could attenuate both FFA-induced cell death and inflammation in NASH.

Both DR5 and its ligand, TRAIL, are up-regulated in the liver of human patients with NASH [56]. TRAIL knockout mice are protected from diet-induced NASH in a murine model [48]. However, DR5 signaling during lipoapoptosis has been shown to be independent from its ligand TRAIL [36]. Interestingly, though, TRAIL is included in EVs released during lipotoxic DR5 signaling (Fig. 2). TRAIL-containing EVs activate DR5 on the surface of macrophages, leading to increased expression of the anti-inflammatory cytokines interleukin (IL)-1β and IL-6 (Fig. 2) [48]. These findings support the existing non-canonical role for TRAIL as a pro-inflammatory mediator [57]. Finally, the release of pro-inflammatory EVs depends on Rho GTPases, a family of serine/threonine kinases that contribute to various cellular events, including vesicle trafficking (Fig. 2) [58]. Notably, the Rho-kinase (ROCK) inhibitor fasudil hydrochloride hydrate (Fasudil) is frequently used in Japan to treat subarachnoid hemorrhage and prevent cerebral vasospasm and subsequent ischemic injury [59]. Thus, repositioning of fasudil may result in an effective treatment for EV-induced inflammation in NASH (Fig. 2).

## c-Jun N-terminal kinase (JNK) 1 plays a central role in NASH

**Fig. 3:**
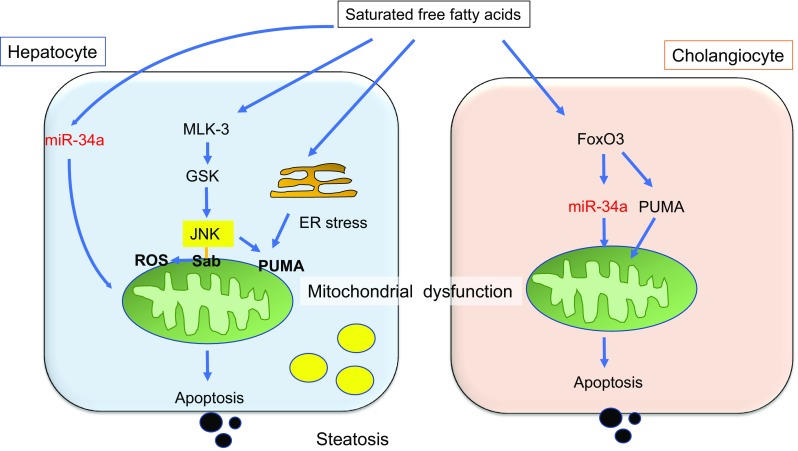
Lipotoxicity in hepatocytes and cholangiocytes. In hepatocytes, mixed lineage kinase 3-glycogen synthase kinase 3β (MLK-3-GSK)-activated c-Jun N-terminal kinase (JNK) phosphorylation plays a central role in apoptosis. JNK and endoplasmic reticulum (ER) stress protein CHOP cooperatively induce the BH3-only protein PUMA, leading to apoptosis. JNK binds to Sab, an mitochondrial outer membrane protein, which promotes translocation of JNK to mitochondria, inducing reactive oxygen species (ROS). MiR-34a also contributes to lipoapoptosis. Although saturated free fatty acids (FFAs) are poorly incorporated into lipid droplets, steatosis occurs to some extent in hepatocytes. In contrast, FFAs do not induce steatosis in cholangiocytes. Lipotoxicity is mediated by forkhead box O3 (Foxo3)-stimulated miR-34a and PUMA up-regulation. JNK does not contribute to cell death in cholangiocytes

JNKs are serine/threonine kinases belonging to the mitogen-activated protein kinase (MAPK) family [60]. JNK activation by saturated FFAs plays a central role in lipoapoptosis and the pathogenesis of NASH, as well as obesity and insulin resistance [18, 61–63]. Saturated, but not unsaturated FFAs, induce JNK activity in cultured cells (Fig. 3) [19]. There are three isoforms of JNK: JNK1, JNK2, and JNK3. Although hepatocytes express JNK1 and JNK2, the saturated FFA-induced lipoapoptosis and pathogenesis of NASH appears to depend on JNK1 [61]. Signaling upstream of JNK in lipoapoptosis involves mixed lineage kinase 3 (MLK-3) and glycogen synthase kinase 3β (GSK3β) (Fig. 3) [64, 65]. In addition, several JNK inhibitors have already been tested in clinical trials for other diseases, including idiopathic pulmonary fibrosis and inflammatory endometriosis [60]. Further development of JNK1 isoform-selective inhibitors may, therefore, be beneficial for the treatment of obesity and NASH.

Another recent study examined caspase 8 and FADD-like apoptosis regulator (CFLAR) as a mediator of JNK signaling in NASH. This discovery was somewhat surprising as CFLAR is a well-known negative regulator of above-mentioned receptor signaling [35]. Hepatocyte-specific *Cflar* knockout in HFD-fed mice promoted increased body and liver weights and led to a more severe version of NASH that included inflammatory changes in the liver. HFD-induced changes in JNK1 activation were reversed by hepatic *Cflar* overexpression. The study also determined that CFLAR likely inhibited the MAP3K5/ASK-1/JNK1 pathway and it should be noted that an ASK-1 inhibitor is already being tested in a clinical trial for the treatment of NASH [66]. This study also used primate models to demonstrate that increasing CFLAR expression via a liver-targeted therapeutic gene vector attenuated symptoms associated with HFD-induced NASH, including fibrosis. The authors concluded that CFLAR-peptide-mimicking drugs could be beneficial for the treatment of NASH [67]. Although the CFLAR/ASK-1/JNK1 pathway may contribute to inflammation and fibrosis during NASH, the ASK-1-related pathway may be dispensable for hepatocyte cell death, as ASK-1 inhibition has been shown to have no effect on palmitic acid-induced JNK1 activation and apoptosis [68].

## Autophagy and endoplasmic reticulum (ER) stress in NASH

Autophagy is an intracellular pathway responsible for the turnover of unwanted proteins or organelles [69, 70] and it can also regulate intracellular lipid levels by removing lipid droplets through a process termed lipophagy [71]. Although autophagy serves as a quality control mechanism for organelles and proteins, dysregulation of autophagy can promote cell death [72]. Dysregulation of autophagic function has also been reported to promote the development of NASH and contribute to hepatocyte lipoapoptosis [73]. The current understanding is that although FFAs can induce initiation of autophagy, they inhibit autophagic flux, defined by the rate of autophagic degradation [74]. Palmitic acid-induced inhibition of autophagic flux results in the accumulation of p62, an increase in the microtubule-associated protein 2 light chain 3 (LC3-II):LC3-I ratio, and accumulation of autophagosomes [73]. In fact, p62 levels are significantly higher in the liver of patients with NASH compared to those with NAFL [73]. Pharmacological promotion of autophagic activity by carbamazepine and rapamycin has also been shown to improve a murine model of NASH [75]. However, this finding raises the question as to how FFAs impair autophagic flux. To this end, a recent study demonstrated that palmitic acid impaired autophagy flux by preventing the late stages of autophagy. Miyagawa et al. demonstrated fusion of autophagosomes and lysosomes in cultured hepatocytes after saturated FFA treatment, independently of lysosomal functions of acidification and hydrolysis [76]. Impairment of autophagic flux induced the accumulation of autophagosomes in palmitic acid-treated but not oleic acid-treated cells. In addition, Tanaka et al. investigated the association between NASH and rubicon, a beclin 1-interacting negative regulator of autophagosome–lysosome fusion [77]. Rubicon is post-transcriptionally up-regulated by palmitic acid, suppressing the late stages of autophagy. Inhibition of rubicon by RNAi restored palmitic acid-induced autophagy impairment as shown by reduction of p62 and LC-II, which lead to reduced apoptosis In addition, mice with hepatocyte-specific rubicon knockout displayed improvements in liver steatosis and injury and restored autophagic function. Rubicon deficiency was shown to inhibit JNK signaling both in vitro and in vivo. Accordingly, this protein may serve as a key mediator of the two major death pathways during lipotoxicity, and targeting it may contribute to the treatment of NASH. Interestingly, JNK may activate p62 via an as-yet undefined mechanism in leukemia cells [78]; it should thus be determined whether, conversely, JNK-induced signals could alter autophagy in NASH. Another recent study suggested that medium-chain fatty acids might restore rubicon-suppressed autophagy in HFD-induced NASH [79]. Therefore, one potentially beneficial option may be to replace a portion of the saturated FFAs in a diet with medium-chain fatty acid-containing products, such as coconut oil.

**Fig. 4:**
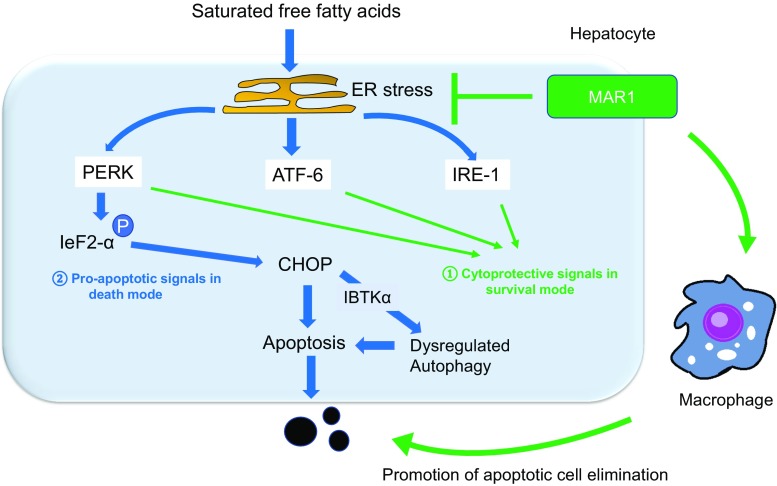
Endoplasmic reticulum (ER) stress-mediated apoptosis. Saturated free fatty acids (FFAs) induce ER stress, activating protein kinase R-like ER kinase (PERK), inositol requiring 1 (IRE1), and activating transcription factor 6 (ATF6). These signals first activate the protective unfolded protein response (UPR); however, excessive stress leads to activation of apoptotic signaling, mainly through PERK-mediated CHOP up-regulation. miR-615-3p, which suppresses CHOP expression, is decreased during FFA treatment. Augmentation of miR-615-3p levels partially inhibits CHOP expression and cell death induced by FFA-mediated ER stress. Maresin 1 (MAR1) resolves lipotoxicity and ER stress by up-regulating UPR pro-survival mechanisms and preventing the excessive stimulation of pro-apoptotic pathways. MAR1 is also able to attenuate ER stress in macrophages, restoring Kupffer cell phagocytic capacity to clear apoptotic hepatocytes

The ER plays an essential role in homeostasis by regulating cellular protein folding and assembly. Disruption of ER homeostatic mechanisms by toxic reagents or nutrient excess induces the accumulation of misfolded or unfolded proteins within the organelle. This triggers the unfolded protein response (UPR), leading to ER stress [80]. ER stress is triggered by ER transmembrane sensors that include protein kinase R-like ER kinase (PERK), inositol requiring 1 (IRE1), and activating transcription factor 6 (ATF6) (Fig. 4). During the UPR, these sensors are released from the intraluminal chaperone glucose-regulated protein 78 (GRP78) [81–83]. The UPR initially transmits signals throughout the cell to inhibit protein synthesis and increase the capacity of the ER. However, when stress overwhelms ER capacity, the pathway shifts to transmitting pro-death signals, inducing apoptosis (Fig. 4) [84]. ER stress has been linked to various pathological conditions, including NASH [85]. Multiple studies have demonstrated the up-regulation of ER stress markers in NASH. For example, ATF6 is up-regulated in NASH livers compared to normal tissues [86], and the levels of GRP78 and GRP94, ER chaperone proteins involved in cell survival during UPR, are significantly downregulated in patients with NASH [86]. Several studies have also examined the mechanisms of saturated FFA-induced ER stress and cell death [61, 87–89]. These studies have revealed that the ER stress-induced pro-apoptotic protein DNA damage inducible transcript 3 (DDIT3)/CHOP is a major player in ER stress-mediated hepatocyte lipoapoptosis, and it has been extensively studied in this context [87, 88]. CHOP is at least partially activated by phosphorylation of eukaryotic Initiation Factor 2 (eiF2) alpha downstream of PERK (Fig. 4). CHOP and above-mentioned JNK cooperatively activate BH3 only protein PUMA to induce mitochondrial dysfunction and cell death (Fig. 3) [87]. Although IRE1 can induce JNK activation during ER stress, it may not contribute to cell death during FFA-induced apoptosis [64, 65, 68, 90].

Recent ER stress-related research has shed light on some newly determined mediators of active cell recovery from stress, despite the previous belief that healing from stress after injury was a passive event [91]. Maresin 1 (MAR1) is one of the various specialized pro-resolving lipid mediators that has been found to actively facilitate the return of injured tissue to homeostasis [91]. Rius et al. showed that MAR1 reduced saturated FFA-induced apoptosis and stress caused by the ER stress inducer tunicamycin by regulating the UPR response in hepatocytes (Fig. 4) [92]. In addition, MAR1 increased phagocytosis in Kupffer cells, promoting the clearance of apoptotic hepatocytes. Because MAR1 was isolated as a pro-resolving mediator whose activity is promoted by omega-3 essential FAs, this study potentially explains how polyunsaturated FFAs exert their positive effects on obesity-related diseases. It also suggests the possibility of efficiently applying specific polyunsaturated FFA-derived mediators beneficial to patients with NASH.

Current data suggest that ER stress and autophagy are not independent phenomena but are interconnected. For example, autophagy is induced to dispose of misfolded proteins remaining after ER-associated protein degradation [93]. Recently, Willy et al. investigated an isoform of inhibitor of Burton’s tyrosine kinase (IBTKα) [94], a component of the UPR that resides mainly in the ER and is preferentially translated during ER stress [95].They showed that IBTKα was up-regulated downstream of PERK-CHOP pathway during palmitate-induced ER stress [95], which was associated with aberrant autophagy linking to consequent inhibition of autophagic flux (Fig. 4). Loss of IBTKα enhanced survival of human hepatocytes. Optimal amount of IBTKα, therefore, plays a newly discovered role in FFA-induced autophagy initiation, via the ER.

## Reactive oxygen species (ROS)

Mitochondria are also a target of FFA assault. During NAFLD, mitochondria try to adapt to increased FFA flux by increasing rates of β-oxidation; however, increased transport of β-oxidation-derived products to the mitochondrial electron transport chain (ETC) leads to increased ROS release [96]. Although hepatocytes have antioxidant defenses to scavenge ROS, the balance in NAFLD is generally shifted towards ROS production [96]. Excessive production of ROS is believed to induce oxidative stress, leading to further impairment of mitochondrial respiration. Treatment of hepatocytes with palmitic acid results in a concentration-dependent stimulation of β-oxidation-induced respiration. However, above a threshold of palmitic acid, mitochondrial respiration becomes gradually impaired, followed by release of cytochrome *c* to the cytosol leading to apoptosis [90, 97, 98]. Increased production of ROS and decreased antioxidant activity are indeed observed in human NAFLD as well as in animal models of steatohepatitis. Butylated hydroxyanisole (BHA), an antioxidant, counteracts lipotoxicity in cultured rat hepatocytes [97] and vitamin E has been shown to be superior to a placebo for the treatment of biopsy-proven NASH in human adults without type 2 diabetes [99].

Regarding recent developments in ROS-related research, interesting interactions between JNK and mitochondrial respiration have been reported [97]. Interaction of JNK with Sab, an outer membrane mitochondrial protein, leads to inhibition of mitochondrial respiration during palmitic acid treatment and increased ROS release, thus contributing to cell death in cultured hepatocytes (Fig. 3) [97]. Sab knockdown significantly inhibited palmitic acid-induced cell death in cultured hepatocytes. Interestingly, this effect occurred only at the late stage of apoptosis, suggesting that Sab-related JNK activity contributed to cell death via gradual impairment of mitochondrial dysfunction. [97]. Another interesting study showed that cytochrome *c* was released from mitochondria to the cytosol of ob/ob mice challenged with an HFD (a relatively early model of NAFLD), but did not result in caspase 3/7 activation or apoptosis [100]. However, the mitochondria derived from the liver of these NAFLD model mice presented an altered size and were more susceptible to Ca^2+^-induced permeability transition, as well as the entry of water and small molecules. Early cytochrome *c* release was related to the alteration of a complex that consisted of the phosphorylated voltage-dependent anion channel, Bcl-xl, and GSK3β on the outer mitochondrial membrane, which in the normal state prevents the release of cytochrome *c* [100]. This study suggested a sensitization toward the mitochondrial pathway of apoptosis even in early phases of NAFLD.

In addition, an intriguing role of transforming growth factor (TGF)-β in ROS production has been reported recently [101]. TGF-β, a well-known factor responsible for the formation of fibrotic scar tissue in the liver, was shown to participate in ROS production and hepatocyte death in lipid-accumulated hepatocytes [101]. Expression of TGF-β receptor type I was shown to be increased in lipid-accumulated hepatocytes and, furthermore, co-treatment of palmitic acid and TGF-beta synergistically increased ROS production and cell death in cultured rat hepatocytes, which was reversed by the antioxidant BHA. These results indicated that lipid-accumulated hepatocytes potentiated TGF-β-mediated ROS production and possibly contributed to cell death. Thus, TGF-β may play a role not only in fibrosis formation, but also during the early stages of NAFLD.

## Epigenetic factors and hepatocyte lipotoxicity: novel targets

Epigenetic changes are defined as chemical modifications of genomic DNA that are unrelated to alteration of the primary DNA sequence. They include DNA methylation, altered expression of non-coding RNAs, histone modification, and chromatin remodeling [102, 103]. Epigenetic changes can be reversed by interventional approaches [104], raising clinical interest in this area [105–107]. This chapter will mainly focus on non-coding RNAs, the most intensively investigated form of epigenetic machinery and the one most related to hepatocyte death signaling [108].

Non-coding RNAs do not encode proteins, but function as cellular signaling modulators that regulate gene expression as well as protein translation. MicroRNAs (miRNAs) are small non-coding RNAs (19–23 nucleotides) that modulate RNA functions as well as post-transcriptional regulation of gene expression. Variety of miRNAs are found to be either up-regulated or down-regulated in human NASH [103], diet-induced animal models of NASH [109], and free fatty acid-treated hepatocytes [110]. For example, miR-200a, miR-200b, miR-200c, miR-146a, miR-146b, and miR-152 were shown to be up-regulated during FFA acid-treated human heoatpcytes as well as in high-fat diet-fed mice model [110]. In human liver biopsy, dozens of miRNAs were found to be differentially expressed in NASH compared to normal controls [103], including mir-122 and mi-192. In addition to the miRNAs in the heopatocytes, circulating miRNAs in the serum are stable and protected from RNAase-mediated degradation; thus, they are extensively studied as potential biomarkers of NAFLD. Variations of miRNAs, including miR-122, miR-192, mir-19a, miR-19b, miR-125b, and miR-375, were shown to significantly up-regulated in the serum in both NAFL and NASH [111]. However, miR-122 level was significatnly higher in NASH compared to NAFL [111].  Further, recent study identified a panel composed of miR-122-5p, miR-1290, miR-37-3p, miR-192-5p that showed high diagnostic accuracy to identify NAFLD [112],

Majority of miRNAs implicated in NAFLD are reported in affect lipid metabolism [113, 114] and some others are indicated in inflammation. In terms of hepatic lipotoxicity, targeting these miRNAs could potentially decrease the amount of FFAs in hepatocytes which, in turn, might reduce the risk of hepatocyte apoptosis. Of all the miRNAs, liver-specific miR-122 is believed to modulate key players of lipid metabolism including FAS, HMGCR, SREBP-1c and SREBP-2 [103], and mice with conditional deletion of miR-122 develop steatohepatitis. MiR-122 has been shown to correlate with CK-18 levels in the serum of NASH patients. Thus, miR-122 may serve as a candidate marker for disease severity [111].

Recently, miR-34a has emerged as a key regulator of hepatic lipid homeostasis and has gained attention because of its significance in metabolic diseases [111, 115–118]. MiR-34a levels were reportedly up-regulated in steatotic hepatocytes as well as in liver tissues of HFD-fed mice, and are associated with disease severity in the liver of human NAFLD patients [113, 115]. Overexpression of miR-34a has been shown to increase lipid accumulation as well as FFA-induced apoptosis in cultured primary rat hepatocytes (Fig. 3) [115]. A recent functional study suggested that inhibition of miR-34 directly targeted peroxisome proliferator-activated receptor-α (PPARα), an essential modulator of lipid transport and metabolism of the β-oxidation pathway, resulting in decreased TG content in hepatocytes [113]. Cholestatic presentation of NASH has been suggested to develop in a subset of patients [119] and FFAs induce apoptosis not only in hepatocytes but also in cholangiocytes. Indeed, cholangiocyte lipoapoptosis has been shown to occur in high fat-high sucrose-fed mice [117, 120]. Natarajan et al. recently found that FFAs induced apoptosis of cholangiocytes through forkhead box O3 (Foxo3)-mediated miR-34a (Fig. 3). Foxo3a has also been found to promote expression of the pro-apoptotic BH3 protein PUMA, which has been shown to trigger apoptosis in cholangiocytes (Fig. 3). In hepatocytes, the above-mentioned JNK activates PUMA in cooperation with ER-stress-induced CHOP, inducing mitochondrial dysfunction and cell death. Interestingly, in contrast to hepatocytes, cholangiocytes do not die following JNK activation; thus, Foxo3a-stimulated PUMA and miR-34 may play critical roles in cholangiocytes (Fig. 3) [117]. Another difference of palmitate-induced change between cholangiocytes and hepatocytes is steatosis. Although palmitic acid is poorly incorporated into lipid droplets, steatosis occurs to some extent in hepatocytes. In contrast, saturated free fatty acids do not seem to induce steatosis in cholangiocytes. These differences might result from different expression of proteins that modulate lipid synthesis, liphophagy, and lipolysis: however, further studies are required. 

Another recent study focused on miRNAs that regulate metabolic ER stress-induced apoptosis [121]. Miyamoto et al. found decreased miR-615-3p in hepatocytes treated with palmitic acid and tunicamycin, an ER stress inducer. They found that augmentation of miR-615-3p levels partially inhibited CHOP mRNA expression and cell death (Fig. 4). They noted that decrease of miR-615-3p was induced by ER stress, but was independent of eiF2alpha phosphorylation. Thus, their results suggested that up-regulation of miR-615-3p could be a novel approach to treat NASH.

In addition to miRNAs, long non-coding RNAs (lncRNAs), defined as transcripts longer than 200 nucleotides, are increasingly recognized as having a potential role in NAFLD. Genome-wide studies have revealed over 500 lncRNAs to be down-regulated, and over 1200 lncRNAs to be up-regulated in the liver of NAFLD patients [108]. One of the most profound discoveries was by Atanasovska et al., who recently found a novel liver-specific lncRNA, nc18q22.2, on chromosome 18, which showed significantly elevated expression in the liver tissue of NASH patients. In addition, knockdown of lnc18q22.2 in cultured hepatocyte cell lines resulted in either reduced cell growth or cell death [122]. The authors could not detect any apoptosis or an increase of biochemical markers such as cleaved PARP, which suggested caspase 3-independent hepatocyte death. Nevertheless, apoptosis in these knockdown cells could not be entirely ruled out, as 18q22.2 seemed to regulate anti-apoptotic BH3-only proteins such as Mcl-1. The study of non-coding RNAs has thus provided new insights into the regulation of hepatocyte viability in NASH and widens the possibility of intervention.

## Die another way? Caspase-independent death signals and NASH

Although apoptosis is the fundamental process by which organized cell death occurs, caspase inhibition does not completely inhibit hepatocyte cell death in NASH [32]. This has led researchers to consider other types of cell death [123–125]. Treatment with palmitic acid induces not only apoptosis, but also an emerging type of cell death termed necroptosis [123, 124]. Although categorized as a form of programmed cell death, necroptosis does not utilize caspases but rather receptor-interacting proteins (RIP) 1 and 3 and the phosphorylation of mixed lineage kinase domain-like (MLKL) proteins [126, 127]. The decision to undergo apoptosis or necroptosis seems to depend on interactions between caspase 8 and RIP1/RIP3 [128]. Necroptosis may, therefore, serve as a backup pathway to enable cell death when apoptosis is inhibited and vice versa.

RIP3 has been found to be elevated in models of NASH, but not NAFL [123], and FFA treatment does appear to induce necroptosis. Thus, RIP3 activation and necroptosis may actually be present in NASH. However, recent functional studies with RIP3 knockout mice have provided controversial results. Although RIP3 knockout mice were protected from NASH induced by an MCD diet [123, 124], HFD-induced liver injury and steatosis were exacerbated in RIP3-deficient mice [125]. Although both diets induce similar pathological features, their pathogenic mechanisms are quite distinct. For example, MCD diets do not induce insulin resistance, whereas HFD-induced NASH exhibits insulin resistance similar to human liver disease. In contrast, an MCD diet is able to induce much more prominent inflammation in the liver compared to a HFD alone. It, therefore, seems that the effect of RIP3 depends on the metabolic state of the liver and that inhibiting RIP3 does not seem practical for insulin resistance-driven NASH. Further studies employing other mediators of necroptosis, such as MLKL, should ideally be performed to explore this concept.

Finally, new forms of cell death, such as ferroptosis (the iron-dependent accumulation of lipid hydroperoxides) [129] and pyroptosis (another inflammatory form of programmed cell death mediated by human caspases 1, 4 and 5) [130], have been recently described in other diseases. However, the role of these new types of cell death in NASH remains unclear and will again require further investigation.

## Conclusions

**Fig. 5:**
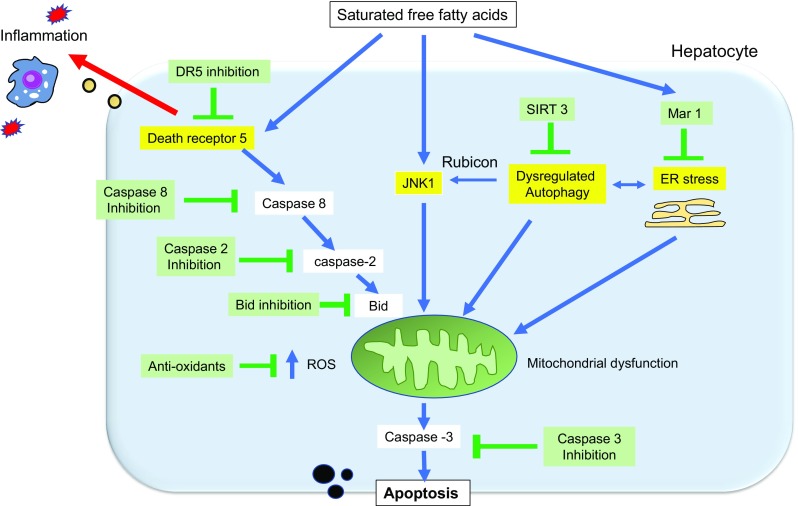
Interactions between lethal and nonlethal pro-inflammatory signaling by saturated free fatty acids (FFAs) and potential interventions for NASH. Saturated FFAs induce apoptotic signaling through multiple pathways, including endoplasmic reticulum (ER) stress, death receptor (DRS) and c-Jun N-terminal kinase (JNK) signaling, non-coding RNAs, reactive oxygen species (ROS), and dysregulation of autophagy. These signals ultimately merge to induce mitochondrial dysfunction and the release of the executioner caspase 3, leading to cell death. Sublethal amounts of FFAs induce pro-inflammatory signaling in parenchymal cells, leading to inflammation and fibrosis. Based on recent discoveries regarding pathways involved in FFA-induced toxicity, the proteins and drugs highlighted in green are potential interventional targets for NASH

Pro-apoptotic signaling in NASH involves multiple mediators, such as JNK, DR5, ER stress, autophagy, and ROS. These pathways form a signaling network (Fig. 5) that leads hepatocytes to either complete apoptosis, or to survive with injury. Recent advances suggest that even when apoptosis is incomplete, FFA-induced cell death signals are harmful for the progression of NASH. This is due to the induction of pro-inflammatory and pro-fibrotic signals to neighboring parenchymal cells. Targeting of apoptotic signaling may, therefore, inhibit hepatocyte cell death, as well as inflammation, in NASH patients.
